# Impact of queuosine modification of endogenous *E. coli* tRNAs on sense codon reassignment

**DOI:** 10.3389/fmolb.2022.938114

**Published:** 2022-08-31

**Authors:** Jillyn M. Tittle, David G. Schwark, Wil Biddle, Margaret A. Schmitt, John D. Fisk

**Affiliations:** Department of Chemistry, University of Colorado Denver, Denver, CO, United States

**Keywords:** genetic code expansion, sense codon reassignment, tRNA modification, queuosine modification, synthetic biology, orthogonal translation machinery

## Abstract

The extent to which alteration of endogenous tRNA modifications may be exploited to improve genetic code expansion efforts has not been broadly investigated. Modifications of tRNAs are strongly conserved evolutionarily, but the vast majority of *E. coli* tRNA modifications are not essential. We identified queuosine (Q), a non-essential, hypermodified guanosine nucleoside found in position 34 of the anticodons of four *E. coli* tRNAs as a modification that could potentially be utilized to improve sense codon reassignment. One suggested purpose of queuosine modification is to reduce the preference of tRNAs with guanosine (G) at position 34 of the anticodon for decoding cytosine (C) ending codons over uridine (U) ending codons. We hypothesized that introduced orthogonal translation machinery with adenine (A) at position 34 would reassign U-ending codons more effectively in queuosine-deficient *E. coli*. We evaluated the ability of introduced orthogonal tRNAs with AUN anticodons to reassign three of the four U-ending codons normally decoded by Q34 endogenous tRNAs: histidine CAU, asparagine AAU, and aspartic acid GAU in the presence and absence of queuosine modification. We found that sense codon reassignment efficiencies in queuosine-deficient strains are slightly improved at Asn AAU, equivalent at His CAU, and less efficient at Asp GAU codons. Utilization of orthogonal pair-directed sense codon reassignment to evaluate competition events that do not occur in the standard genetic code suggests that tRNAs with inosine (I, 6-deaminated A) at position 34 compete much more favorably against G34 tRNAs than Q34 tRNAs. Continued evaluation of sense codon reassignment following targeted alterations to endogenous tRNA modifications has the potential to shed new light on the web of interactions that combine to preserve the fidelity of the genetic code as well as identify opportunities for exploitation in systems with expanded genetic codes.

## 1 Introduction

The incorporation of an amino acid into a growing peptide chain is the ultimate outcome of a series of competition events. Amino acids, tRNAs, and aminoacyl tRNA synthetases (aaRSs) identify their cognate partners to appropriately charge each tRNA. In the ribosome, each mRNA codon is sampled by the complement of aminoacylated tRNAs in the cell. Following recognition between the codon-anticodon pair, the amino acid may be incorporated into the growing protein chain. Thermodynamic interactions interpreted through kinetic decisions by the ribosome and elongation factors enable a cognate tRNA to progress through the successive steps of translation more efficiently than a non-cognate tRNA (Rodnina and Wintermeyer, 2001; Fluitt et al., 2007).

Expansion of the genetic code via incorporation of noncanonical amino acids (ncAAs) in response to sense or stop codons is the outcome of the same set of competition events, complicated by the addition of orthogonal translation machinery (tRNA/aaRS pair). Increasing the efficiency of codon reassignment by an orthogonal pair may be achieved by altering one or more of the steps in the progression from tRNA aminoacylation to peptide bond formation in the ribosome. A significant barrier to broadly expanding the genetic code is the incomplete quantitative understanding of the factors that control the fidelity of the genetic code, e.g. codon-anticodon interaction energies, aminoacylation efficiency, relative tRNA concentrations, and the influence of tRNA modifications on each of these components. As the genetic code is engineered to allow codon-specific incorporation of multiple copies of multiple ncAAs into a single protein, the ability to improve orthogonal pair competition by modulating precise functions of the endogenous translation machinery will become increasingly important.

The fidelity of protein translation is the extent to which the “correct” tRNA successfully incorporates its charged amino acid and “incorrect” tRNAs are rejected at some point along the path. Misincorporation of amino acids into proteins occurs at a rate of approximately 1 error in 1,000 to 10,000 peptide bond forming events (Parker, 1989; Kramer and Farabaugh, 2007). While missense errors are thought to be generally destabilizing, missense mutations induced by antibiotic treatment, defective aaRS editing, or sense codon reassignment are broadly tolerated by cells (Min et al., 2003; Ruan et al., 2008; Schmitt et al., 2018). The missense error rate is a function of the complement of translational machinery present in the cell. Missense errors differ across codons in the same cell. Missense errors at individual codons differ with growth rate and across organisms (Parker, 1989; Kramer and Farabaugh, 2007).

In *E. coli*, tRNA species with 40 anticodon sequences decode the 61 sense codons. Most tRNA species are tasked with decoding more than a single sense codon. The pairing of tRNA anticodon to mRNA codon is often mediated by modified nucleotides in the tRNA. *E. coli* is one of the few organisms for which the complete set of tRNA modifications and modifying enzymes have been identified. *E. coli* tRNA species contain, on average, 8 modified bases each, nearly 12% of the entire tRNA molecule (El Yacoubi et al., 2012; Björk et al., 2014). The anticodon stem loop is the most highly and most diversely-modified region of tRNAs. Within the anticodon, position 34, which interacts with the third/wobble position of the mRNA codon, may be modified to one of 14 different non-UCAG nucleotides. tRNA modifications serve multiple functions in translation, including modulating the tRNA-protein, tRNA-mRNA, and tRNA-ribosome interactions that combine to determine the fidelity of translation.

Certain position 34 tRNA anticodon modifications serve to expand the space of recognition to codons not typically decoded, while other modified anticodons restrict decoding to prevent missense errors (reviewed in: El Yacoubi et al., 2012; Björk et al., 2014). *E. coli* tRNAs with uridine at position 34 (U34) feature two types of modifications, largely determined by whether the tRNA is part of a four-codon box (one amino acid for all codons in the box) or a two-codon box (U3/C3 codons encode one amino acid; A3/G3 codons encode another). In most four boxes, uridine-5-oxyacetic acid at position 34 allows a single tRNA to decode the expected A3 and G3 codons as well as the U3 codon. In two boxes, 5-methylaminomethyl-2-thiouridine at position 34 restricts decoding to the A3 and G3 codons only because the U3 codon encodes a different amino acid. A single *E. coli* tRNA, tRNA^Arg2^_ICG_, includes modification of A34 to inosine. The inosine modification expands anticodon-codon recognition beyond the expected A34/U3 base pairing and allows tRNA^Arg2^ to decode the U3, C3, and A3 codons. A single *E. coli* tRNA, tRNA^Ile2^_LAU_, is transcribed as C34 and subsequently modified to lysidine. The lysidine modification restricts decoding to the Ile AUA codon only (Lysidine 34/A3 pairing). A U34 tRNA with a UAU anticodon would be expected to also decode the Met AUG codon via a U34/G3 wobble pairing. L34 does not pair with G3 (El Yacoubi et al., 2012; Björk et al., 2014).

**FIGURE 1:**
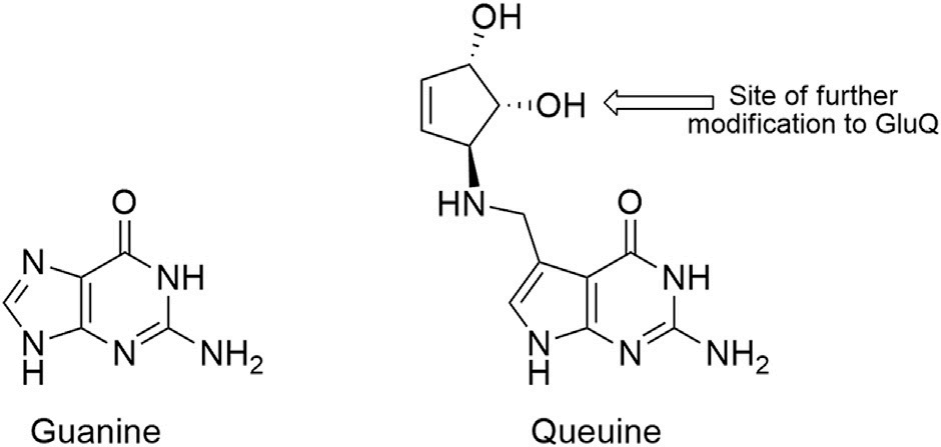
Structures of guanine (G, left) and the modified guanine, queuine (Q, right). The arrow on queuine indicates the position of glutamic acid esterification, a further modification found in the *E. coli* aspartic acid tRNA.

The extent to which alteration of endogenous tRNA modifications may be exploited to improve genetic code expansion efforts has not been broadly investigated. Despite having important evolutionarily conserved functions, the vast majority of *E. coli* tRNA modifications are not essential. Of the 29 different modifications encoded by 50 genes, only 4 are essential (6 of 50 genes) (Datsenko and Wanner, 2000; Kitagawa et al., 2005). We identified queuosine (Q, Figure 1), a non-essential, hypermodified guanosine nucleoside found in position 34 of the anticodons of four *E. coli* tRNAs as an anticodon modification that could potentially be exploited to improve sense codon reassignment (Bienz and Kubli, 1981; Dineshkumar et al., 2002). The four NAU/NAC codon pairs [tyrosine (UAU/UAC), histidine (CAU/CAC), asparagine (AAU/AAC), and aspartic acid (GAU/GAC)] are each decoded by a single tRNA species tRNA_QUN_ in *E. coli*. The encoded guanosine at position 34 of each of these tRNAs is replaced by queuosine, one of the larger and more complex nucleotide modifications (Björk et al., 2014).

Eight gene products are involved in queuosine biosynthesis and tRNA modification (Iwata-Reuyl, 2003; Björk et al., 2014). Five enzymes are responsible for the synthesis of the 7-methylaminodeazaguanine precursor base, queuine (queuine is the modified base, queuosine is the modified nucleotide). An enzyme catalyzes the exchange of the queuine base for the genetically-encoded guanine base in appropriate tRNAs. Two additional enzymes function to further decorate the 7-methylamino group of queuine with a ribose-derived cyclopentenediol ring (Figure 1). In aspartic acid tRNAs (QUC anticodon), queuosine is further modified to GluQ by addition of a glutamic acid residue (Salazar et al., 2004). Queuosine modification has been identified in tRNAs having QUN anticodons across most organisms, save yeast, archaea, and *Thermus thermophilus*. Prokaryotes have the ability to synthesize queuine from guanosine. Eukaryotes strip queuine from the anticodons of bacterial tRNAs acquired either through their diet or, in higher organisms, from gut microbiota.

Early studies on queuosine modifications in translation suggest that a primary function may be to reduce the C3 over U3 codon bias of G34 tRNAs. Comparisons of tRNA-tRNA anticodon parings suggested that Q34-C3 pairings were less energetically favorable that G34-C3 pairings while Q34-U3 pairings were more stable than G34-U3 pairings (Grosjean et al., 1978). In *E. coli*, queuosine-modified tyrosine tRNAs appear to sample the ribosome twice as fast as unmodified tRNAs (Noguchi et al., 1982). Evaluation of the *in vivo* decoding properties of Drosophila histidine tRNAs in Xenopus ooctyes suggested that unmodified G34 tRNAs prefer C3 codons while Q34 tRNAs equally decode C- and U-ending codons (Meier et al., 1985). tRNA identity, reading frame maintenance, and prevention of missense errors are additional functions in protein translation ascribed to queuosine modification (Giege et al., 1998; Urbonavicius et al., 2001; Manickam et al., 2014; Manickam et al., 2016). Beyond the direct evaluations of tRNA modifications affecting decoding properties in natural genetic codes, differential tRNA modifications have been proposed as a general cellular response to stress and as a component of the development of cancer and other disease states (Chen and Wu, 1994; Nagaraja et al., 2021). Queuosine modification of tRNAs has been shown to vary across developmental stages of Drosophila (Zaborske et al., 2014).

**FIGURE 2:**
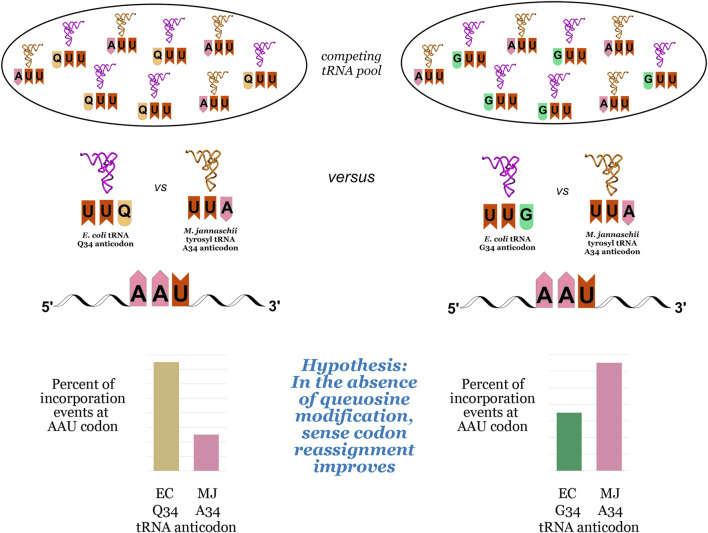
Competition events for decoding NAU codons in *E. coli*, using the asparagine AAU codon as an example. The pool of competing tRNAs includes the orthogonal *M. jannaschii* tRNA aminoacylated with tyrosine and the endogenous tRNAs with either queuosine (Q) at position 34 in the anticodon (left) or guanosine (G) at position 34 in the anticodon (right). The pair of bar graphs at the bottom of the figure illustrate the hypothesis that an orthogonal tRNA would more efficiently compete against G34 endogenous tRNAs than Q34 endogenous tRNAs, leading to increased efficiency of sense codon reassignment (SCR).

We hypothesized that the inherent bias of G34 tRNAs for C-ending codons could be exploited to improve the reassignment of U-ending codons (Figure 2). The introduction of an orthogonal tRNA capable of Watson-Crick base pairing to the U-ending codon in queuosine-deficient strains could lead to increased U-ending codon reassignment in two ways: first, by taking advantage of the energetic favorability of orthogonal tRNA A34/U3 base pairing relative to the endogenous G/Q34/U3 wobble, and second, by reducing the efficiency of competition from the endogenous tRNA G34/U3 wobble relative to the Q34/U3 wobble. We evaluated the effects of queuosine knockout on the reassignment efficiency of the *Methanocaldococcus jannaschii* (*M. jannaschii*) tyrosyl tRNA/aaRS orthogonal pair at three of the four U-ending codons read by Q34 *E. coli* tRNAs using our previously-described fluorescence-based screen (Biddle et al., 2015). As our screen relies on differentiating tyrosine incorporation from the naturally encoded amino acid, we are unable to evaluate the tyrosine (UAU/UAC) codon pair.

Prior studies on the effect of queuosine modification in protein translation reveal divergent effects across organisms and among the subset of queuosine-modified tRNAs within a single organism. Our findings bear out these observations. Orthogonal tRNAs with AUN anticodons in queuosine modification-deficient cells exhibit sense codon reassignment efficiencies that are slightly improved at Asn AAU, equivalent at His CAU, and less efficient at Asp GAU codons relative to the parent, non-queuosine-deficient strain. Utilization of an introduced orthogonal pair in our screen also allowed evaluation of base pairing competition events that are not present within standard genetic codes. We found that an orthogonal tRNA whose A34 anticodon is partially modified to inosine competed much more effectively against the queuosine-deficient *E. coli* tRNA^His^_GUG_ than tRNA^His^_QUG_ for decoding the His CAU codon.

## 2 Methods and materials

The supplementary materials file includes detailed experimental protocols for the previously-published fluorescence-based screen. The file also includes general reagents and materials, cell strain details, and a procedure for the preparation of electrocompetent cells (Sambrook and Russell, 2001).

### 2.1 Principle of the fluorescence-based screen

**FIGURE 3:**
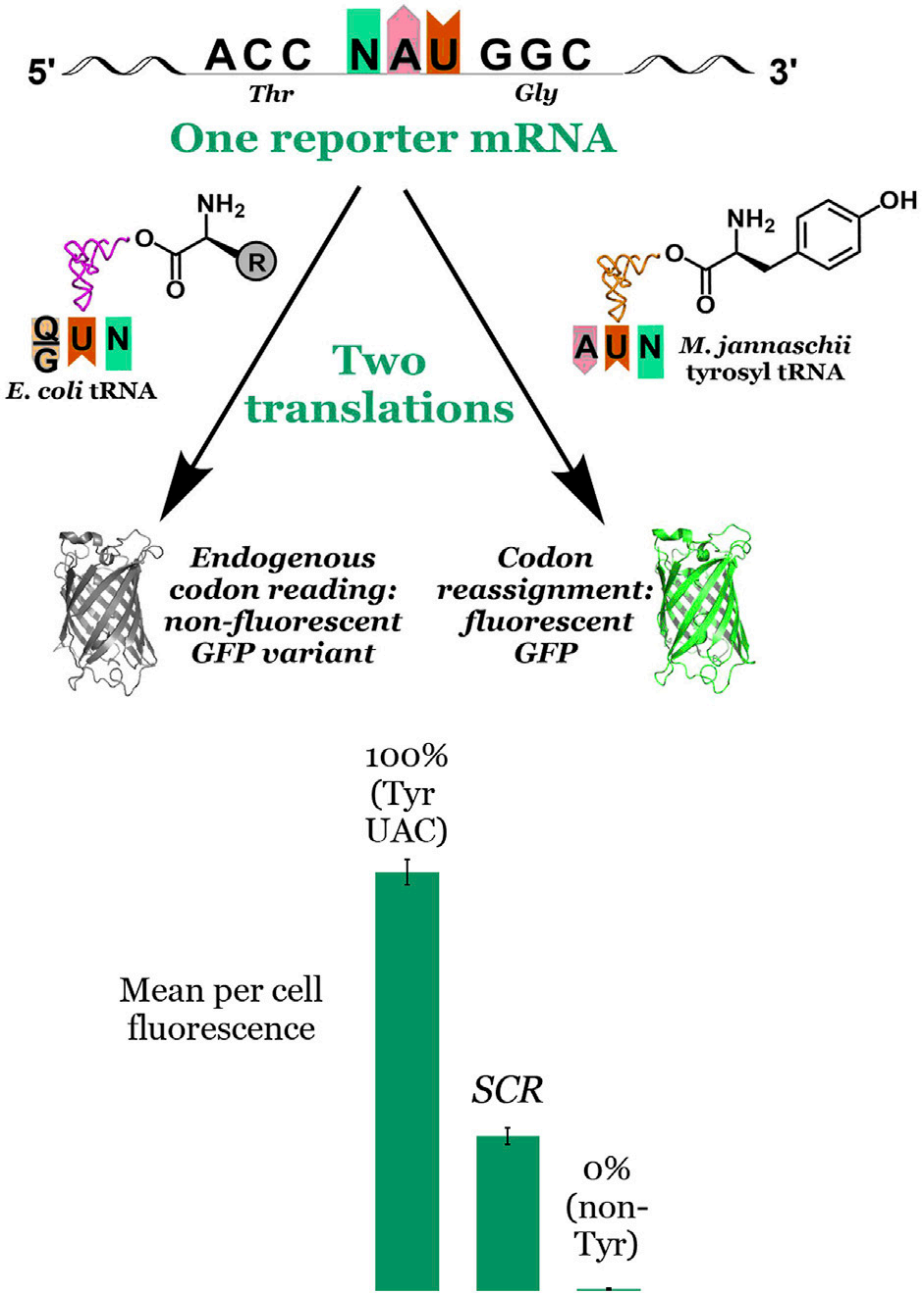
Principle of and data visualization for the fluorescence-based screen for codon reassignment. The fluorescence-based screen exploits the absolute requirement of tyrosine at position 66 in superfolder GFP for fluorophore formation. Reporter vectors with another codon at that position are co-transformed with the *M. jannaschii* tyrosyl aminoacyl tRNA synthetase and a variant of its cognate tRNA. Both the engineered orthogonal tRNA and endogenous tRNAs (with either Q or G at position 34) compete to decode the evaluated codon. Incorporation of tyrosine by the orthogonal tRNA in response to the codon at position 66 in the GFP reporter leads to production of fluorescent protein. Incorporation of a different canonical amino acid by the endogenous translation machinery leads to production of non-fluorescent protein. The mean per cell fluorescence of the system under evaluation is bracketed between a 100% fluorescence reference system in which the codon at position 66 encodes tyrosine and a 0% fluorescence reference system in which the codon at position 66 encodes another amino acid. The middle bar labelled “SCR” represents the intermediate per cell fluorescence observed for a hypothetical system under evaluation. In this report, SCR efficiencies range from 2.1% to 13.4% depending on the system. More broadly, we have reported SCR efficiencies between 1.0% and 70% using the *M. jannaschii* orthogonal pair.

Unlike stop codon suppression in which missed incorporations by an orthogonal pair lead to truncated proteins, sense codon reassignment (SCR) results in a heterogeneous mixture of full length proteins. As such, SCR efficiency cannot be readily measured by the yield of full length protein. Rather, the efficiency of incorporation of the amino acid on the orthogonal tRNA relative to that by endogenous tRNAs must be determined via another metric. Our in cell, fluorescence-based screen exploits the absolute requirement of tyrosine at position 66 in green fluorescent protein (GFP) for fluorophore formation. The assay evaluates the extent to which an introduced orthogonal aaRS capable of charging tyrosine to its cognate tRNA is able to reassign the meaning of a test codon placed at the essential fluorophore Tyr position in GFP (Figure 3). The observed per cell fluorescence represents a direct measurement of the orthogonal tRNA’s ability to compete against endogenous tRNAs to decode the targeted codon. Sense codon reassignment efficiencies distill down to a single data point the complex interplay of levels of orthogonal and endogenous tRNAs, the interactions between the orthogonal aaRS and its cognate tRNA, and differences in codon-anticodon interaction energies between the introduced and endogenous tRNAs.

### 2.2 Considerations for screen deployment and reporter gene sequence design

Codon reassignment efficiency is quantified by bracketing the measured per cell fluorescence of each system under evaluation between the measured per cell fluorescence of 0% and 100% reassigning control systems. The 100% fluorescence reference is established in each experiment by monitoring the per cell fluorescence of a reporter vector with a tyrosine codon specifying the fluorophore. The 0% fluorescence reference is established in each experiment by monitoring the per cell fluorescence of a reporter vector with a non-tyrosine codon specifying the fluorophore. Both control systems also include a vector expressing a variant of the orthogonal translation machinery to maintain a metabolic burden on the cells similar to that of the systems under evaluation for reassignment efficiency.

To reduce variation across reassignment systems, the DNA sequences for the reporter vectors for each targeted codon are identical, save the codon at the critical tyrosine position (test position). The gene sequence of the GFP reporter utilizes a reduced codon set to minimize the possibility that reassignment of a particular sense codon at a non-permissive position would interfere with protein folding and fluorophore formation, resulting in a lower apparent SCR efficiency for that codon. The reporter vectors utilized in this evaluation are those used in our broad evaluation of the reassignment potential of more than 30 rarely-used and/or *E. coli* wobble codons (Schmitt et al., 2018; Schwark et al., 2020). Supplementary Figure S1 depicts the codon usage frequency for the wild type superfolder GFP gene used in the 100% fluorescence reference system. The nearly identical sequences of the two vectors used across all evaluations should minimize apparent reassignment differences as a result of different rates or levels of mRNA transcription/codon context effects.

### 2.3 Chromosomal knockout of a key gene in the queuosine biosynthetic pathway, QueC

QueC knockout strains were prepared in house according to the method of Datsenko and Wanner (Datsenko and Wanner, 2000). The method employs a two-step process in which the lambda red recombination system (beta, gam, and exo gene products) facilitates the recombination of an introduced linear DNA sequence in place of a targeted gene, followed by flp-mediated recombination to remove the initially-introduced selective marker. The linear DNA segment is prepared by amplification of a cassette containing a selectivity marker flanked by frp recombinase sites. The amplification primers introduce sequences identical to the sequence flanking the chromosomal region to be removed. The linear DNA is introduced into cells containing a temperature-sensitive plasmid expressing the lambda red system. After selection for recombination and plasmid loss, a second temperature-sensitive plasmid containing the flp recombinase is introduced into the cells, and the initial selective marker is removed. Chromosomal knockout of specified genes was confirmed by sequencing purified PCR products which amplified relevant regions of the chromosome. Sequences of the oligonucleotides used to affect and confirm the genomic knockouts are listed in the supplementary material (Supplementary Table S1).

## 3 Results

Our fluorescence-based screen (Figure 3) rapidly generates single point measurements of the outcome of the multiple competition events that are involved in the incorporation of a given amino acid in response to a given codon. An orthogonal tRNA/aaRS pair, in this case the *M. jannaschii* tyrosyl tRNA/aaRS pair, is introduced into a cell harboring a vector encoding a GFP reporter in which the codon for the essential Tyr residue at position 66 has been replaced by a codon under evaluation. The orthogonal tRNA is aminoacylated with tyrosine by the orthogonal aaRS. Fluorescent GFP is produced when the orthogonal tRNA decodes the codon at position 66 in the GFP reporter vector. Non-fluorescent protein is produced when the endogenous translation machinery decodes the test codon. Bracketing the observed per cell (i.e., normalized to optical density) fluorescence in the system under interrogation with systems in which no fluorescent GFP is produced (0% reassignment control) and one in which wild type superfolder GFP is produced (100% reassignment control) provides a quantitative measurement of the efficiency of reassignment of the particular codon by the orthogonal tRNA variant. In the lower section of Figure 3, the middle bar labelled “SCR” represents the intermediate per cell fluorescence observed for a hypothetical system under evaluation. In this report, SCR efficiencies range from 2.1% to 13.4%. Other anticodon variants of the *M. jannaschii* orthogonal pair facilitate sense codon reassignment to tyrosine with efficiencies between 1.0% and 70% (Schmitt et al., 2018; Schwark et al., 2020).

Most sense codon reassignment evaluations reported by our laboratory have been performed in *E. coli* SB3930, a histidine auxotroph (Biddle et al., 2015; Biddle et al., 2016; Schmitt et al., 2018; Schwark et al., 2020). Our first evaluation of the effect of queuosine modification of endogenous tRNAs on codon reassignment by an orthogonal tRNA looked at reassignment of His CAU in SB3930 and SB3930/QueC-. We chose to employ knockouts of the QueC gene because spontaneous QueC deletions have been shown to eliminate queuosine modification of tRNAs without inducing growth defects (Dineshkumar et al., 2002; Gaur and Varshney, 2005). The QueC gene is involved in the initial steps of the queuosine biosynthetic pathway in *E. coli* (Gaur and Varshney, 2005; El Yacoubi et al., 2012). Removal of queuosine modification does not affect other tRNA modifications (Manickam et al., 2016). Reassignment efficiency of the His CAU codon improved more than 50% from 6.3 ± 0.4% in SB3930 to 10.1 ± 0.7% in SB3930/QueC-.

We proceeded with further evaluations of the effects of queuosine modification on sense codon reassignment in the more frequently-used laboratory strain *E. coli* Top10 and derivatives. The observed reassignment efficiency of His CAU was 7.4 ± 0.2% in Top10 to 11.5 ± 0.3% in Top10/HisB-/QueC-. Differences in incorporation efficiency between strains of *E. coli* despite using identical vectors and system conditions (e.g., media, antibiotics) have been widely observed though not holistically evaluated. In order to confirm that any observed changes in sense codon reassignment efficiency between Top10 *E. coli* and a Top10 strain incapable of modifying tRNAs with queuosine were the result of the change to the queuosine pathway as opposed to the histidine biosynthetic pathway (a notable difference between SB3930 and Top10), we evaluated the sense codon reassigning systems in 3 *E. coli* strains: Top10, Top10/HisB-, and Top10/HisB-/QueC-. Every codon evaluated showed identical reassignment efficiencies in both Top10 and Top10/HisB-, suggesting that reassignment efficiency in rich media is independent of the strain’s ability to synthesize its own histidine. Reassignment efficiencies in Top10/HisB- are provided in the supporting information.

We evaluated the ability of introduced orthogonal tRNAs with AUN anticodons to reassign three of the four U-ending codons normally decoded by Q34 endogenous tRNAs: His CAU, Asn AAU, and Asp GAU in the presence and absence of queuosine modification (Table 1). The reassignment efficiency of the asparagine AAU codon improved modestly (1.2-fold) in the absence of queuosine. In the case of aspartic acid, however, removal of the queuosine modification resulted in a 1.6-fold *decrease* in reassignment of the GAU codon. *E. coli* tRNA^Asp^_QUC_ is hypermodified at position 34 beyond the addition of queuosine; a glutamic acid residue is esterified to the dihydroxycyclopentene ring to form a GluQ nucleoside. Very little is understood about the function of GluQ in translation. Previous evaluations of the effects of queuosine modification on frameshift maintenance did not evaluate aspartic acid codons (Urbonavicius et al., 2001). Evaluations of the effect of queuosine modification on missense incorporation indicated that the absence of queuosine manifested different effects for different tRNAs (Manickam et al., 2014; Manickam et al., 2016).

**TABLE 1:** Sense codon reassignment efficiency (SCR) by variants of the *M. jannaschii* orthogonal translation machinery with AUN anticodons in queuosine-containing and queuosine-deficient *E. coli*.

Orthogonal anticodon	Codon evaluated	Reassignment efficiency in Top10	Biological replicates Top10	Reassignment efficiency in Top10/HisB-/QueC-	Biological replicates Top10/HisB-/QueC-	Fold change in queuosine-deficient strain
AUU	AAU (Asn)	7.5 ± 0.5%	18	8.9 ± 0.4%	11	1.2
	AAC (Asn)	B.D^a^	18	B.D.	16	—
AUC	GAU (Asp)	3.0 ± 0.2%	18	2.1 ± 0.2%	12	0.7
	GAC (Asp)	B.D.	18	B.D.	17	—
AUG/IUG	CAU (His)	7.4 ± 0.2%	24	11.5 ± 0.3%	12	1.6
	CAC (His)	2.8 ± 0.2%	22	2.6 ± 0.1%	11	1.0
AUG G37	CAU (His)	13.2 ± 0.4%	24	13.4 ± 0.8%	15	1.0
	CAC (His)	B.D.	24	B.D.	17	—

a B.D., indicates that the codon was evaluated with the specified tRNA, and the measurement was below the detection limit of the in cell assay (0.15%).

Sense codon reassignment of histidine CAU codons utilized two variants of the orthogonal tRNA. We previously demonstrated that the *M. jannaschii* tRNA_AUG_ is partially (∼50%) modified to inosine in *E. coli* and that anticodon loop modifications outside the anticodon could eliminate the partial inosine modification (Biddle et al., 2016). Both the A34/I34 mixed population tRNA as well as *M. jannaschii* tRNA_AUG-G37_ in which no inosine-modification is detected were evaluated. The partially inosine-modified orthogonal tRNA competed much more effectively in the queuosine-deficient strain, displaying a 1.6-fold improvement in reassignment efficiency. The non-inosine-modified orthogonal tRNA reassigned the CAU codon equally well in both strains.

Importantly, neither sense codon reassignment nor the removal of queuosine modification from GUN anticodon endogenous tRNAs is fatal to cells. QueC chromosomal knockouts have previously been reported to not show large growth defects (Dineshkumar et al., 2002; Gaur and Varshney, 2005). The limited effect of QueC knockout on cell growth is consistent with our observation of similar optical density vs. time profiles of the same codon reassignment system in Top10 and Top10/HisB-/QueC- strains. Our previous evaluations of sense codon reassignments have revealed that *E. coli* are largely tolerant of widespread missense incorporation of tyrosine across their proteomes (Schmitt et al., 2018; Schwark et al., 2020). While the carrying capacity of sense codon reassigning systems is slightly reduced relative to the nonreassigning controls, none of the systems exhibits large growth defects. Representative OD600 vs. time plots for systems expressing all four evaluated *M. jannaschii* tRNAs and their targeted GFP reporters are provided in the supplementary material (Supplementary Figure S2). The supplementary material also includes representative fluorescence versus time plots for the controls and codon reassigning systems in both the queuosine-containing and queuosine-deficient strains (Supplementary Figure S3). The lack of queuosine does not have a significant impact on protein production. Similar fluorescence for the 100% control (GFP with a tyrosine codon at the fluorophore) is observed in both *E. coli* strains.

## 4 Discussion

### 4.1 Could engineering endogenous tRNA modifications serve as a handle for improving the function of expanded genetic code systems?

Increasing the efficiency of codon reassignment by an orthogonal pair may be achieved either by *improving* the function of the orthogonal pair in the system into which it is transplanted or by *decreasing* the efficacy of the endogenous translation components competing for the targeted codon. Improving the function of the orthogonal pair may be as simple as increasing the effective concentrations of orthogonal components by expressing both tRNAs and aaRSs from cassettes with stronger promoters in plasmids with increased copy numbers (Ryu and Schultz, 2006; Young et al., 2010; Chatterjee et al., 2013). Adjustments to media composition, including adjusting amino acid concentrations, have been utilized to improve codon reassignment (Dougherty et al., 1993; Budisa et al., 1995; Kwon et al., 2003; Pal and Budisa, 2010). Increasing the assimilation of the orthogonal machinery into the endogenous translation system may also increase the efficiency of codon reassignment. Amber stop codon suppression has been improved by altering nucleotides on the tRNA that modulate interactions with elongation factor Tu (EF-Tu) (Guo et al., 2009; Schrader et al., 2011; Mittelstaet et al., 2013; Fan et al., 2015; Maranhao and Ellington, 2017).

Improving the interactions between the orthogonal tRNA and its cognate synthetase is another means by which the function of the orthogonal tRNA may be increased. Orthogonal tRNA/aaRS recognition becomes particularly relevant as the anticodon of the orthogonal tRNA is changed to target different sense codons. The anticodon sequence is often an important identity element for proper recognition and aminoacylation of tRNAs (Giege et al., 1998). Poor charging of an amino acid onto its cognate tRNA lowers the effective concentration of aminoacylated tRNA available to compete against endogenous components for a targeted codon. The impacts of changes to anticodon sequences on the efficiency of aminoacylation have been evaluated for the *M. jannaschii* Tyr tRNA/aaRS pair, one of the two orthogonal pairs most commonly used for incorporation of ncAAs in response to amber stop codons (Fechter et al., 2001). Changing the anticodon of the *M. jannaschii* tRNA from GAU (Tyr) to Watson-Crick base pair with other codons decreases aminoacylation efficiency over a range of three orders of magnitude. Several selection strategies have been developed to improve the function of stop codon suppressing orthogonal pair systems, although the maturation of initially-identified functional aaRSs utilized for ncAA incorporation are not commonly performed (Pott et al., 2014; Wang et al., 2015; Rauch et al., 2016; Maranhao and Ellington, 2017; Owens et al., 2017). Directed evolution of *M. jannaschii* tRNA/aaRS pair variants for improved reassignment of the amber stop codon as well as Lys AAG, His CAU, and Arg AGG sense codons have been described (Takimoto et al., 2009; Kuhn et al., 2010; Mittelstaet et al., 2013; Biddle et al., 2015; Wang et al., 2015; Biddle et al., 2016; Biddle et al., 2022).

The alternative strategy of *decreasing* competition from the endogenous translation components that compete with the orthogonal machinery has been applied to improve amber stop codon reassignment and rare arginine codon reassignment. Three different strategies of genomic modifications have been described that enable the elimination of the normally essential peptide release factor, RF1, that recognizes the amber UAG codon as a stop signal. Systems from which RF1 has been eliminated show improved incorporation of ncAAs in response to UAG codons (Johnson et al., 2011; Lajoie et al., 2013; Mukai et al., 2015). Reassignment of the low frequency Arg AGG codon in *E. coli* has been demonstrated after knocking out one of the two competing tRNA genes (Mukai et al., 2015; Lee et al., 2016). Additional genome rewriting projects seek to generate multiple “free” codons for ncAA incorporation (Ostrov et al., 2016; Wang et al., 2016; Robertson et al., 2021). A free codon is one that is not employed in any genes in the organism and does not have a tRNA to decode it. Total genome synthesis and transplantaion are costly and experimentally demanding and may have potential unintended consequences on organismal viability as a result of inadvertant disruptions to regulatory sequences or RNA folding.

An alternative and not widely-explored approach is to modulate the modifications of tRNA species that affect their decoding properties. Functions for tRNA modifications based on their locations in tRNAs and roles that modified tRNAs play in translation have been broadly explored but many questions still remain (Agris, 1991; Lim and Curran, 2001; El Yacoubi et al., 2012; Björk et al., 2014; de Crecy-Lagard and Jaroch, 2021). We sought to evaluate the extent that directed changes in queuosine modification of endogenous anticodons at position 34 could be exploited to alter the reading of the genetic code in *E. coli*. Queuosine is thought to bias the codon reading preferences of the endogenous tRNAs, although data is sparse, and the effects appear to differ across organisms and even between modified tRNAs within the same organism (Noguchi et al., 1982; Manickam et al., 2014; Manickam et al., 2016). Unmodified G34 tRNAs efficiently decode C-ending codons through traditional Watson-Crick interactions and wobble to U-ending codons with reduced efficiency. Q34 tRNAs appear to read U- and C-ending codons equally well (Meier et al., 1985).

### 4.2 Do A34 tRNAs compete better against G34 than Q34 tRNAs for U-ending codons?

Evaluation of A34 tRNA competition is complicated by the limited number of Q34 modified tRNAs and the inability of the fluorescence-based screen to evaluate natural tyrosine incorporation. The fluorescence-based screen quantifies incorporation of tyrosine in response to non-tyrosine codons, which excludes evaluation of the impact of queuosine modification on sense codon reassignment of the Tyr UAU codon. Interpreting the quantification of reassignment of the aspartic acid codon GAU is fraught, as the modified nucleotide on aspartic acid tRNAs is the hypermodified variant of Q, GluQ. Finally, we previously identified partial inosine modification of the *M. jannaschii* tRNA_AUG_ used to reassign the His CAU codon. A single nucleotide change at position 37 of the anticodon loop abrogated inosine modification.

Four codon pairs whose decoding is potentially influenced by queuosine modification exist. We evaluated two systems that directly address the competition of A34 orthogonal vs. Q34 or G34 endogenous tRNAs (*M. jannaschii* tRNA_AUU_ for Asn AAU codons and *M. jannaschii* tRNA_AUG-G37_ for His CAU codons). We found that *M. jannaschii* tRNA_AUU_ reassigned the asparagine AAU codon 1.2 times better in the queuosine-deficient strain (7.5 ± 0.5% vs. 8.9 ± 0.4%). We found that *M. jannaschii* tRNA_AUG-G37_ reassigned the histidine CAU codon equally well in either strain (13.2 ± 0.4% and 13.4 ± 0.8%). Sections 4.3, 4.4 elaborate on the evaluation of two additional systems that offer insight into the impact of modified A34 orthogonal or hypermodified Q34 endogenous tRNAs on competition for the targeted codons.

Meier and co-workers demonstrated that Drosophila histidine tRNA isoforms differing only at the identity of the nucleotide at position 34 showed distinct decoding preferences for histidine CAU versus CAC codons (Meier et al., 1985). Tritiated histidine was aminoacylated onto either the G34 isoform or the Q34 isoform. The amount of label incorporated in response to CAU and CAC codons while in competition with the other isoform aminoacylated with unlabeled histidine provided a window on the decoding preferences of the two tRNAs. When the G34 tRNA was labeled, the labeled amino acid was incorporated in response to the CAC codon twice as often as in response to the CAU codon. In contrast, when the Q34 tRNA was labeled, the labeled amino acid was incorporated in response to the CAC and CAU codons nearly equally, with even a slight preference for CAU over CAC.

Urbonavicius and co-workers evaluated the ways in which Q34 and G34 tRNAs differentially interact with the His CAU and CAC codons (Urbonavicius et al., 2001). Employing a frameshifting reporter, they measured the kinetics of entry of the two tRNA isoforms into the ribosomal a site. The likelihood of frameshifting by a P site tRNA increases as the amount of time required to accept a tRNA into the A site increases. Urbonavicius et al. found that when U-ending codons for tyrosine (UAU) and histidine (CAU) were in the ribosomal A site, queuosine-deficient G34 tRNAs had increased sampling time in the ribosomal A site leading to measurable P site frameshifting. In contrast, when the C-ending codons for tyrosine (UAC) and histidine (CAC) were in the A site, queuosine-deficient G34 tRNAs did not increase P site frameshifting. The absence of queuosine modification did not increase frameshifting when the asparagine U-ending codon (AAU) was in the A site.

Both reports concluded that queuosine is more important for efficient translation of the U-ending codons than C-ending codons, particularly for histidine and tyrosine. Additionally, the presence or absence of queuosine modification influences global cellular protein translation and has been implicated in organismal development and several disease states, including cancer (Chen and Wu, 1994; Zaborske et al., 2014; Nagaraja et al., 2021). Genes containing more NAC codons are expressed when queuosine modification is at a low level. Genes containing more NAU codons are expressed as queuosine modification of relevant tRNAs increases (Zaborske et al., 2014). These observations suggest that queuosine deficiency would disfavor reading U-ending codons by endogenous tRNAs and improve codon reassignment by orthogonal tRNAs targeted to U-ending codons. Our quantification of the reassignment efficiencies of asparagine and histidine U3 codons by *M. jannaschii* tRNAs with A34 anticodons, tRNA_AUU_ and tRNA_AUG-G37_ (Table 1), is in line with these observations and suggests that removal of queuosine modification results in a modest improvement in sense codon reassignment.

### 4.3 Evaluation of competition between A34 vs. G34 or A34 vs. GluQ34 in translation

The *E. coli* tRNA that translates aspartic acid codons is further modified at position 34 relative to the other *E. coli* tRNAs with QUN anticodons by esterification of a glutamic acid to the queuosine nucleotide (Figure 1) (El Yacoubi et al., 2012; Björk et al., 2014). Very little is known about the function of the hypermodified GluQ base. Unlike A34 orthogonal tRNA competition that was either modestly improved or unchanged in the absence of queueosine modification, we found that U-ending codon reassignment decreased in efficiency in the absence of GluQ modification. *M. jannaschii* tRNA_AUC_ reassigned the aspartic acid GAU codon only 0.7 times as well in the GluQ-deficient strain (3.0 ± 0.2% vs. 2.1 ± 0.2%).

Neither Meier nor Urbonavicius examined the impact of GluQ modification on translation or frameshifting at aspartic acid U- and C-ending codons. In a series of studies of the effects of tRNA modifications on missense incorporation, Manickam et al. observed different degrees of misreading by *E. coli* tyrosine and aspartic acid tRNAs in the presence and absence of queuosine. The authors employed a series of gain of function enzymatic reporters to evaluate the extent that aspartic acid and tyrosine tRNAs could read alternative codons to restore enzyme function. Their observations suggest that queuosine modification is important for controlling missense errors related to second position mismatches, e.g., tRNA^Asp^_GluQUC_ decoding the glycine GGC codon using a U35/G2 interaction. In queuosine-deficient strains, missense incorporation of aspartic acid in response to Gly GGC increased. In contrast, for another potential U35/G2 interaction, tRNA^Tyr^_QUA_ decoding Cys UGU and UGC codons, missense incorporation of tyrosine decreased in queuosine-deficient strains (Manickam et al., 2016).

The experiments of Manickam et al. evaluated the impact of tRNA modifications on the propensity of tRNAs to incorrectly decode codons for other amino acids as opposed to the effects of modifications on “cognate” codons. Our fluorescence-based gain of function screen evaluates competition between tRNAs that are both (although differently) cognate for the evaluated codon. While not directly comparable, our experiments and those described by Manickam and co-workers suggest that tRNAs modified with Q vs. GluQ impact translation differently. However, the outcome of removing Q vs. GluQ modification on codon recognition at the wobble position appears to be different than the outcome at codons with second position mismatches. At the second position of the codon, removing GluQ modification increases missense incorporation. At the wobble position, removing GluQ modification reduces the efficiency of directed sense codon reassignment.

### 4.4 Evaluation of competition between inosine and queuosine-modified tRNAs

The fluorescence-based screen and other gain of function enzyme systems can be employed to evaluate base pairing competition events that are not present within standard genetic codes in the context of wholly-functional translation systems in living cells. For example, the base pairing preferences of unmodified A34 tRNAs are relatively unknown because in nearly every naturally encoded tRNA, the A34 is enzymatically-modified to inosine. Similarly, the relative strengths of I34/U3 and Q34/U3 base pairing interactions are largely uninvestigated because natural systems do not contain codon boxes in which such competition would exist.

In natural systems, Q34 and I34 tRNAs are only in direct competition in missense situations. One of the two tRNAs would fully base pair with a certain codon while the other tRNA would have a mismatch at position 35 of the anticodon and incorporate an incorrect amino acid. In *E. coli*, this outside-the-box competition could happen between His tRNA QUG and Arg tRNA ICG. In eukaryotes, which utilize I34 tRNAs more extensively in several 4 codon boxes, the instances of Q34 tRNAs potentially competing against I34 tRNAs increases. Manickam and co-workers did not examine the case of *E. coli* tRNA^His^_QUG_, the only one of the four queuosine-containing tRNAs in which a second position misreading error would place a Q34 tRNA (anticodon QUG) in competition with an I34 tRNA (anticodon ICG) for decoding the CGU and CGC arginine codons.

We found that *M. jannaschii* tRNA_AUG_ with partial inosine modification reassigned the histidine CAU codon 1.6 times better in the queuosine-deficient strain (7.4 ± 0.2% vs. 11.5 ± 0.3%). Partial inosine modification of this orthogonal tRNA was identified following observation of unexpected decoding of the histidine CAC codon (Biddle et al., 2016). A34 tRNAs would not be expected to decode C3 codons, whereas I34 tRNAs typically take the place of G34 and U34 tRNAs in their respective boxes. I34 decoding the CAC codon was unaffected by the presence of queuosine in the endogenous *E. coli* tRNA (2.7 ± 0.2% vs. 2.6 ± 0.1% in QueC + vs. QueC- strains). The observation of similar behavior in in QueC + vs. QueC- strains is consistent with the studies suggesting that G34 and Q34 show similar efficiencies for C3 codons. Decoding of the C-ending codons by all other orthogonal tRNAs described in this manuscript is below the limit of detection in both QueC+ and QueC- Top10 *E. coli* (less than 0.15%).

## 5 Conclusion

The relative quantitative contribution of various factors affecting codon-specific translational efficiency are not well understood. Factors including tRNA modifications, tRNA abundance, codon-anticodon interaction energy, codon context effects, interactions between the nascent peptide chain and the ribosome, and interactions between different amino acids in the peptidyl transferase site, among others, all contribute to decoding. The relative importance of the individual factors is codon- and tRNA-dependent. Competition between endogenous and introduced orthogonal tRNAs, integrating all of the above interactions, ultimately determines the efficiency of sense codon reassignment. Our previous quantification of the reassignment efficiency of more than 30 sense codons using the fluorescence-based screen adds to the dissection of the relative quantitative importance of tRNA abundance, aminoacylation efficiency, and codon-anticodon interaction energy as contributors to translational fidelity (Biddle et al., 2015; Schmitt et al., 2018; Schwark et al., 2020).

Our system for evaluating orthogonal pair-directed codon reassignment allows evaluation of the impact of tRNA modifications on the fidelity of translation. Using the fluorescence-based screen and two related *E. coli* strains, one with queuosine-modified tRNAs and one without, the relative efficiencies of five distinct base pairing combinations: A34/U3, G34/U3, Q34/U3, GluQ34/U3, and I34/U3 have been evaluated. These five types of base pairing interactions are analyzed through evaluation of five distinct groups of competition events: A34 orthogonal tRNAs vs. Q34, GluQ34, or G34 endogenous tRNAs or I34 orthogonal tRNAs vs. Q34 or G34 endogenous tRNAs. Quantifying the effect of tRNA modification on decoding specific codons is critical for understanding the overall fidelity of protein translation and for engineering systems with expanded genetic codes.

Removal of queuosine modification of endogenous tRNAs leads to a 0.7 to 1.6-fold improvement of sense codon reassignment by *M. jannaschii* orthogonal tRNAs, depending on both the orthogonal tRNA and the codon targeted for reassignment. These observed effects of queuosine modification on sense codon reassignment may be contextualized by examining the extent to which the efficiency of stop codon suppression is modulated through system modifications and directed evolution. Efforts to improve system function by targeting expression levels, tRNA interactions with EF-Tu and aaRSs, as well as the kinetics of aminoacylation typically result in 1 to 10-fold improvement. Improved orthogonal tRNAs for amber codon suppression were evolved based on optimization of interactions with *E. coli* EF-Tu (Guo et al., 2009). The evolved tRNA sequences typically improved incorporation of noncanonical amino acids 1 to 3-fold, although a handful of improvements up to 20-fold were reported. Maturation of orthogonal tRNA/aaRS pairs initially-evolved for specific noncanonical amino acids yields improvements of 1 to 10-fold (Kuhn et al., 2010; Maranhao and Ellington, 2017; Owens et al., 2017; Thyer et al., 2021). The functional improvements in the efficiency of noncanonical amino acid incorporation are the results of smaller, 1 to 5-fold improvements in the kinetic efficiency of orthogonal pairs (Amiram et al., 2015; Rauch et al., 2016). The context of the targeted stop codon also contributes to the efficiency of suppression (Pott et al., 2014; Schwark et al., 2018).

## Data Availability

The raw data supporting the conclusions of this article will be made available by the authors, without undue reservation.
