# Supplementary material for: Impact of queuosine modification of endogenous E. coli tRNAs on sense codon reassignment
Source: Front Mol Biosci. 2022 Aug 31;9:938114. doi: 10.3389/fmolb.2022.938114 (PMC9471426; doi:10.3389/fmolb.2022.938114)
Supplement: Supplementary file 1 [file DataSheet1.pdf]

Supplementary Material

TABLE OF CONTENTS

S.1: Cell strains	2
S.2: General reagents and materials	2
S.3: Preparation of chromosomal knockout strains	3
Table S.1: Oligonucleotide primers used to construct and verify HisB and QueC knockouts	3
S.4: GFP reporter vectors for codon reassignment	4
Figure S.1: Codon usage frequency for the superfolder GFP gene common to all SCR reporters	4
S.5: *M. jannaschii* tRNA/aaRS pair expression vector backbone details and sequences	4
S.6: GFP fluorescence-based codon reassignment assay protocol	5
S.7: Calculation of codon reassignment efficiencies	5
Figure S.2: Representative optical density at 600nm vs time plots for reassignment of NAU codons by *M. jannaschii* tRNAs with AUN anticodons and nonreassigning controls for 0% and 100% fluorescence in strains with and without queuosine modification of AUN anticodons.	6
Figure S.3: Representative fluorescence vs time plots for reassignment of NAU codons by *M. jannaschii* tRNAs with AUN anticodons and nonreassigning controls for 0% and 100% fluorescence in strains with and without queuosine modification of AUN anticodons.	7
Table S.2: Sense codon reassignment efficiencies in Top10 and Top10/HisB- *E. coli*	8

S.1. Cell strains

Top10 (Invitrogen): F⁻ - *mcrA Δ(mrr-hsdRMS-mcrBC) φ80lacZΔM15 ΔlacX74 recA1 araD139 Δ(ara-leu) 7697 galU galK rpsL (StrR) endA1 nupG λ-*

Top10/HisB- (in house): Top10 Δ*his*B

Top10/HisB-/QueC- (in house): Top10 Δ*his*B Δ*que*C

SB3930 (Yale CGSC): *λ⁻, ΔhisB463*

Top10 knockout strains were prepared in house according to the method of Datsenko and Wanner Datsenko, K. A. and B. L. Wanner (2000). One-step inactivation of chromosomal genes in Escherichia coli K-12 using PCR products. *Proc. Natl. Acad. Sci. U. S. A.* 97(12): 6640-6645. DOI: 10.1073/pnas.120163297). Chromosomal knockout of specified genes was confirmed by sequencing purified PCR products which amplified relevant regions of the chromosome. See Section S.3 and Table S.1 for more details on knockout construction.

S.2 General materials and reagents

All restriction enzymes, DNA polymerases, and T4 kinase were purchased from New England Biolabs and used according to the manufacturer's instructions. ATP was purchased from Fisher (BP413-25) and dNTPs were purchased from New England Biolabs (N0447S). DNA isolation was performed using a Thermo Scientific GeneJET plasmid miniprep kit (K0503) according to the manufacturer's protocols. Intermediate cloning steps and PCR products were purified using a Thermo Scientific GeneJET PCR spin kit (K0701).

LB liquid media (per liter: 10 g tryptone, 5 g yeast extract, 5 g NaCl) and LB agar plates with 15 g/L agar (TEKNova, A7777) were used unless otherwise noted. Isopropyl-beta-D-thiogalactoside (IPTG) was purchased from Gold Bio (I2481C5). Spectinomycin (Enzo Life Science, BML-A281) was used at 50 μg/mL to maintain the vectors harboring the tRNA and aaRS genes. Carbenicillin (PlantMedia, 40310000-2) was used at 50 μg/mL to maintain the vectors harboring the GFP reporter gene. All bacterial cultures were grown at 37 °C unless otherwise noted. All liquid cultures were shaken at 225 rpm unless otherwise noted.

Electrocompetent stocks of all strains were prepared in-house according to the method of Sambrook and Russell (J. Sambrook and D. W. Russell *Molecular cloning: a laboratory manual*. **2001**, Cold Spring Harbor Laboratory press). Typical transformation efficiencies for electrocompetent cells produced in this way are 10^9 cfu/μg of supercoiled DNA. All transformations were recovered in SOC (20 g/L tryptone, 5 g/L yeast extract, 10 mM NaCl, 2.5 mM KCl, 10 mM $MgCl_2$, 20 mM glucose) for 1 hour at 37 °C with shaking prior to transfer to media containing appropriate antibiotics and/or inducers as noted.

All oligonucleotides were purchased from Integrated DNA Technologies (Coralville, Iowa, USA). DNA sequencing was performed by Genewiz (Plainfield, NJ, USA).

S.3 Preparation of chromosomal knockout strains

Top10 knockout strains were prepared in house according to the method of Datsenko and Wanner Datsenko, K. A. and B. L. Wanner (2000). One-step inactivation of chromosomal genes in Escherichia coli K-12 using PCR products. *Proc. Natl. Acad. Sci. U. S. A.* 97(12): 6640-6645. DOI: 10.1073/pnas.120163297). Chromosomal knockout of specified genes was confirmed by sequencing purified PCR products which amplified relevant regions of the chromosome.

E. coli strains and plasmids for affecting the chromosomal knockout were acquired from the *E. coli* Genetic Resources at Yale CGSC, The Coli Genetic Stock Center as components of the "8 Strain Wanner Lambda Red Gene Disruption Kit".

Table S.1: Oligonucleotide primers used to construct and verify HisB and QueC knockouts

Oligo name	Oligonucleotide sequence (5' – 3')
hisB-knockout-5'	GAA GAA AGC CAG CGC GTC ATT GAC GCC TTA CGT GCG GAG CAA GTT TGA TGA TTC CGG GGA TCC GTC GAC C
hisB-knockout-3'	GCA GCC GGT ATC AAG GAT CAC CAC GTT CAT TAC AGC ACT CCT TTC GAC GAT GTA GGC TGG AGC TGC TTC G
hisB-verify-5'	CAC TGA AAG AGA TCC CCT GCG TAG AG
hisB-verify-3'	GCA AGT CGA CGC CGT TGC TCT C
queC-knockout-5'	CAA GCC GCA TCC GGC GAC ACC CGG AAT AAT TAC CTC AAC CCG GTT TTC TGT GTA GGC TGG AGC TGC TTC G
queC-knockout-3'	ATC GCA GGC GTT ACC ATA ACG CTT TTA TTG TTC AAG CAG GAT TAT CTA TGA TTC CGG GGA TCC GTC GAC C
queC-verify-5'	GGA AGG GGA ATT GCG CGA AAA G
queC-verify-3'	CGC AAT GAA TAG CTG GTC CGG G

Sequences of oligonucleotides with "knockout" in the name are based on those used to knockout the same genes in the generation of the Keio collection: Baba T, Ara T, Hasegawa M, Takai Y, Okumura Y, Baba M, Datsenko KA, Tomita M, Wanner BL, Mori H. Construction of Escherichia coli K-12 in-frame, single-gene knockout mutants: the Keio collection. Mol Syst Biol. 2006;2:2006.0008. doi: 10.1038/msb4100050. Epub 2006 Feb 21. PMID: 16738554; PMCID: PMC1681482.

Sequences of oligonucleotides with "verify" in the name are locus-specific primers that are used to verify that the targeted genes are knocked out.

S.4 GFP reporter vectors for codon reassignment

Full sequence data for the suite of GFP reporter vectors used in this manuscript has been reported previously:

1) W. Biddle, M. A. Schmitt, and J. D. Fisk, Evaluating Sense Codon Reassignment with a Simple Fluorescence Screen, *Biochemistry*, **2015**, *54*, 7355-7364.
2) M. A. Schmitt, W. Biddle, and J. D. Fisk, Mapping the Plasticity of the *Escherichia coli* Genetic Code with Orthogonal Pair-Directed Sense Codon Reassignment. *Biochemistry*, **2018**, *57*, 2762-2774.
3) D. G. Schwark, M. A. Schmitt, W. Biddle, and J. D. Fisk, The Influence of Competing tRNA Abundance on Translation: Quantifying the Efficiency of Sense Codon Reassignment at Rarely Used Codons, *ChemBioChem*, **2020**, *21*, 2274-2286.

	codon	reporter usage	AA	codon	reporter usage	AA	codon	reporter usage	AA	codon	reporter usage	AA	
U	UUU	1	F	UCU	0	S	UAU	0	Y	UGU	0	C	U
	UUC	11		UCC	6		UAC	9		UGC	2		C
	UUA	3	L	UCA	1		UAA		oc	UGA		op	A
	UUG	0		UCG	1		UAG		am	UGG	1	W	G
C	CUU	0	L	CCU	0	P	CAU	**0**	H	CGU	7	R	U
	CUC	16		CCC	4		CAC	11[a]		CGC	0		C
	CUA	0		CCA	5		CAA	0	Q	CGA	0		A
	CUG	2		CCG	1		CAG	7		CGG	1		G
A	AUU	0	I	ACU	0	T	AAU	**0**	N	AGU	0	S	U
	AUC	11		ACC	12		AAC	13		AGC	1		C
	AUA	0		ACA	4		AAA	19	K	AGA	0	R	A
	AUG	5	M	ACG	1		AAG	0		AGG	0		G
G	GUU	0	V	GCU	0	A	GAU	**0**	D	GGU	0	G	U
	GUC	15		GCC	6		GAC	18		GGC	11		C
	GUA	3		GCA	3		GAA	17	E	GGA	8		A
	GUG	0		GCG	0		GAG	0		GGG	3		G
	U			C			A			G			

Figure S1. Codon usage frequency for the superfolder GFP gene common to all SCR reporters. The gene sequence of the parent GFP reporter used in each evaluation of codon reassignment has been chosen carefully to minimize the utilization of sense codons likely to be targeted for reassignment. Relevant to this report, no instances of the His CAU, Asn AAU, or Asp GAU codons occur in the reporter. The notation of 11 His CAC codons comprises 5 histidine codons within the GFP gene and 6 in the protein purification tag at the 3' end of the gene. A nearly identical gene sequence is utilized in the suite of reporter vectors to minimize the impact of variations in mRNA transcription and translation on measured efficiency of codon reassignment.

S.5 Vector backbone sequence details for the *M. jannaschii* orthogonal translation machinery

The vector backbone from which the *M. jannaschii* tRNA/aaRS variants are expressed for evaluation using the fluorescence-based screen is based on the vector used in our previous evaluations of sense codon reassignment by the *M. jannaschii* tRNA/aaRS orthogonal pair. The sequence of the entire

vector was reported in W. Biddle, M. A. Schmitt, and J. D. Fisk, Evaluating sense codon reassignment with a simple fluorescence screen, *Biochemistry*, **2015**, *54*, 7355-7364. No changes to the vector were made beyond varying the anticodon.

The gene sequence for the *M. jannaschii* $tRNA^{Opt}$ variants (“nnn” is the anticodon, positions 34-36) is:

```
5′ - CCGGCGGTAGTTCAGCAGGGCAGAACGGCGGACTnnnAATCCGCATGGCAGGGGTTCAAATCC
CCTCCGCCGGACCA - 3′
```

$tRNA^{Opt}_{AUG\ G37}$ has mutation A37G and was originally described as “Colony H2” in W. Biddle, M. A. Schmitt, and J. D. Fisk, Modification of orthogonal tRNAs: unexpected consequences for sense codon reassignment, *Nucleic Acids Research*, **2016**, *44*, 10042-10050.

S.6. GFP fluorescence-based sense codon reassignment assays

Superfolder green fluorescent protein (GFP) reporter plasmids were co-transformed with vectors expressing the modified orthogonal translational components into the appropriate strain of *E. coli*. After overnight growth, colonies were picked into 200 µL LB media in a 96 well plate. Cells were grown to at least mid-log phase (usually 8-10 hours) with shaking at 37 °C. Cells were diluted 10-fold into LB media with antibiotics to maintain the plasmids and 1 mM IPTG for induction of both the aaRS and GFP. Assays were performed in a Fluorotrac 200 clear bottom 96 well plate (Greiner 655096) and monitored in a BioTek Synergy H1 or BioTek Synergy Neo 2S plate reader at 37 °C with continuous double orbital shaking. The optical density (OD600) and fluorescence of each well was measured every 15 minutes for at least 15 hours; optical density was measured at 600 nm, and fluorescence was measured with an excitation at 485 nm and detection at 515 nm with a 20 nm band pass.

S.7 Calculation of sense codon reassignment efficiency from optical density and fluorescence readings

For each biological replicate, the relative fluorescence (corrected fluorescence per OD) was calculated for each of the 16 data points gathered between 8 and 12 hours after induction of GFP and the aaRS with IPTG. The 16 relative fluorescence values were averaged to determine the RFU for each sample. That RFU is divided by the average RFU for all biological replicates of the 100% reassigning fluorescence control (wild type GFP) to determine the reassignment efficiency. Sense codon reassignment efficiency for each tRNA anticodon/GFP codon variant pair was calculated by averaging the reassignment efficiency for at least six biological replicates. All reported codon reassignment efficiencies represent the mean and standard deviation of at least 12 biological replicates as indicated in the main text (Table 1).

An extremely detailed discussion and workflow for calculating codon reassignment efficiency was provided in the Supporting Information for M. A. Schmitt, W. Biddle, and J. D. Fisk, Mapping the Plasticity of the *Escherichia coli* Genetic Code with Orthogonal Pair-Directed Sense Codon Reassignment. *Biochemistry*, **2018**, *57(19)*, 2762-2774.

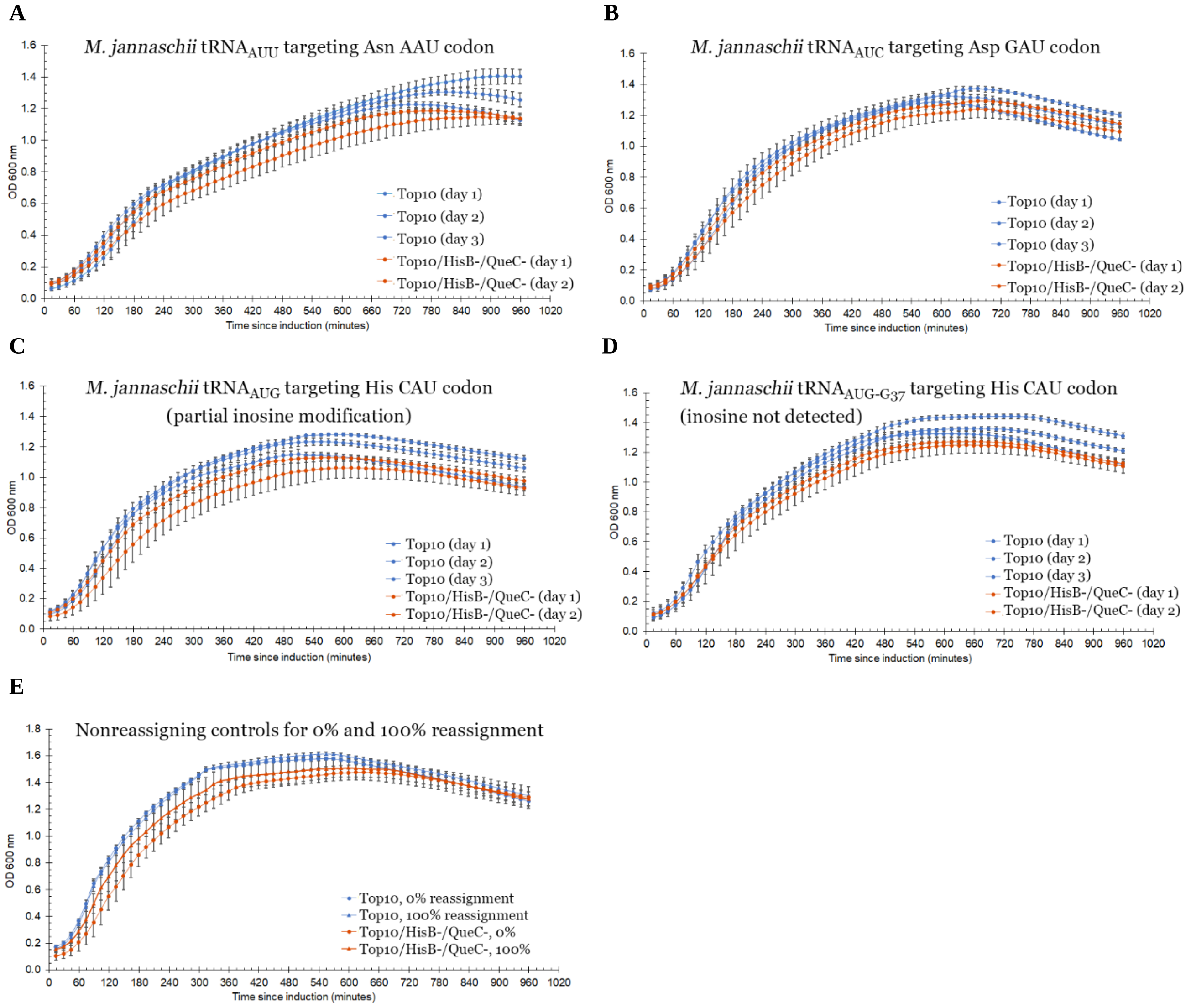

Figure S.2: Representative optical density at 600nm vs time plots for reassignment of NAU codons by *M. jannaschii* tRNAs with AUN anticodons and nonreassigning controls for 0% and 100% fluorescence in strains with and without queuosine modification of AUN anticodons. In parts A-D (sense codon reassignment systems), average growth profiles for 5 representative experiments are shown. Three (blue markers) are for reassignment evaluated in Top10. Two (orange markers) are for reassignment evaluated in Top10/HisB-/QueC-. Each line reflects the average and standard deviation of 5-6 biological replicates for each system. A) *M. jannaschii* tRNA$_{AUU}$ and Asn AAU reassignment reporter. B) *M. jannaschii* tRNA$_{AUC}$ and Asp GAU reassignment reporter. C) *M. jannaschii* tRNA$_{AUG}$ and His CAU reassignment reporter (partial inosine modification of A34 orthogonal tRNA). D) *M. jannaschii* tRNA$_{AUG-G37}$ and His CAU reassignment reporter (no detectable inosine modification of A34 orthogonal tRNA). E) Representative growth versus time profiles for the two nonreassigning control systems included in every evaluation of sense codon reassignment, 0% reassignment (nonfluorescent GFP position 66 reporter) and 100% (GFP reporter with Tyr codon at position 66). Blue circles depict the 0% reassignment reporter evaluated in Top10. Blue triangles depict the 100% reassignment reporter evaluated in Top10. Orange circles depict the 0% reassignment reporter evaluated in Top10/HisB-/QueC-. Orange triangles depict the 100% reassignment reporter evaluated in Top10/HisB-/QueC-. Each line shows the average and standard deviation of 5-6 biological replicates.

A

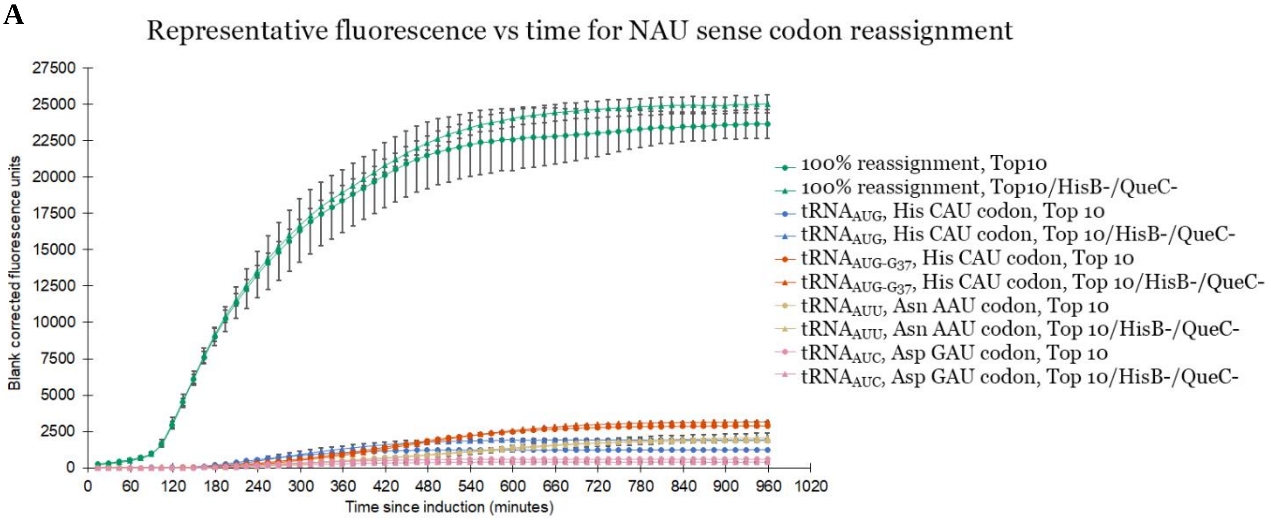

B

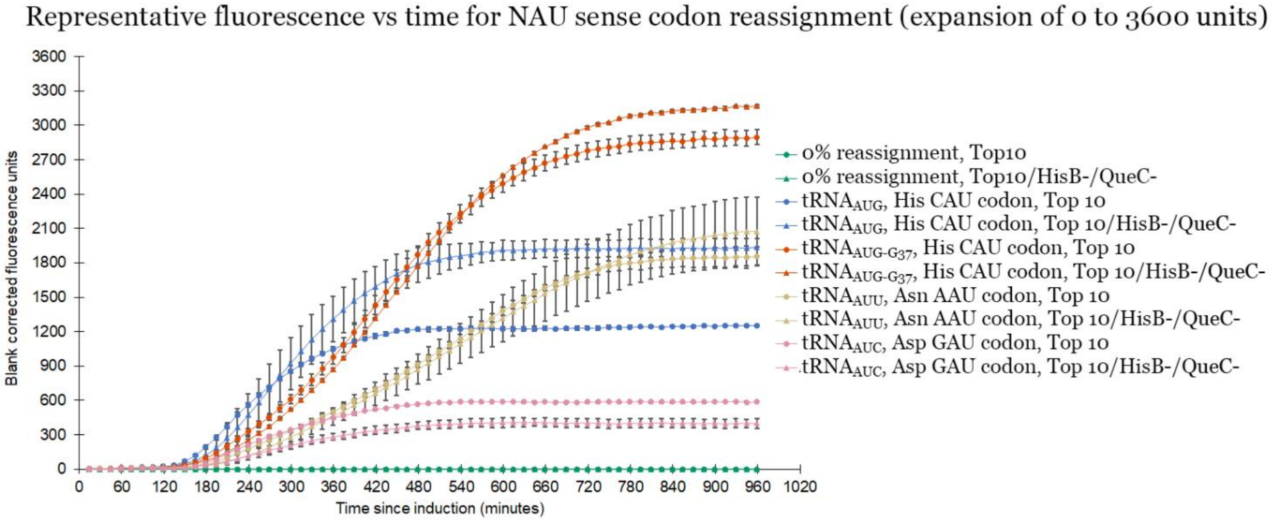

Figure S.3: Representative fluorescence vs time plots for reassignment of NAU codons by *M. jannaschii* tRNAs with AUN anticodons and nonreassigning controls for 0% and 100% fluorescence in strains with and without queuosine modification of AUN anticodons. Each line plots the average and standard deviation of 5-6 biological replicates for each system. Error bars are present on all data points. In some cases, error bars are obscured by the marker. A) Four sense codon reassignment systems and the 100% fluorescence superfolder GFP control in *E. coli* Top10 and Top10/HisB-/QueC-. B) Four sense codon reassignment systems and the 0% fluorescence superfolder GFP control in *E. coli* Top10 and Top10/HisB-/QueC-. Part B expands the y-axis of the graph in part A to highlight those systems with fluorescence between 0 and 3600 fluorescence units (all four SCR systems and the 0% reassignment control). Fluorescence traces are corrected for the changes in media fluorescence that occurs as cells grow and divide. The data shown is not the optical density adjusted fluorescence which is used to determine codon reassignment efficiency. Legend: Circles depict data from Top10 *E. coli*. Triangles depict data from Top10/HisB-/QueC- *E. coli*. Blue lines/markers depict the *M. jannaschii* $tRNA_{AUG}$ and His CAU reassignment reporter (partial inosine modification of A34 orthogonal tRNA). Orange lines/markers depict the *M. jannaschii* $tRNA_{AUG\text{-}G37}$ and His CAU reassignment reporter (no detectable inosine modification of A34 orthogonal tRNA). Gold lines/markers depict the *M. jannaschii* $tRNA_{AUU}$ and Asn AAU reassignment reporter. Pink lines/markers depict the *M. jannaschii* $tRNA_{AUC}$ and Asp GAU reassignment reporter. Green lines/markers indicate the 100% fluorescence reporter (A) and 0% fluorescence reporter (B).

Table S.2: Sense codon reassignment efficiencies in Top10 and Top10/HisB- *E. coli*

Orthogonal anticodon	Codon evaluated	Reassignment efficiency in Top10	Biological replicates: Top10	Reassignment efficiency in Top10/HisB-	Biological replicates: Top10/HisB-
AUU	AAU (Asn)	7.5 ± 0.5%	18	7.5 ± 0.4%	21
	AAC (Asn)	B.D.	18	B.D.	24
AUC	GAU (Asp)	3.0 ± 0.2%	18	3.0 ± 0.1%	18
	GAC (Asp)	B.D.	18	B.D.	18
AUG/IUG	CAU (His)	7.4 ± 0.2%	24	7.4 ± 0.2%	18
	CAC (His)	2.8 ± 0.2%	22	2.7 ± 0.2%	17
AUG G37	CAU (His)	13.2 ± 0.4%	24	13.0 ± 0.2%	12
	CAC (His)	B.D.	24	B.D.	18

B.D. indicates that the reassignment efficiency was below the detection limit of the fluorescence-based screen, 0.15%.

One difference between *E. coli* SB3930, the strain used in most of the laboratory's systematic evaluations of sense codon reassignment efficiency, and the standard laboratory strain Top10 is that SB3930 cells cannot synthesize their own histidine. Since availability of histidine could impact the incorporation of tyrosine in response to histidine codons by lowering the effective concentration of aminoacylated endogenous $tRNA^{His}_{QUG}$, we evaluated the impact of histidine auxotrophism on sense codon reassignment. Every codon evaluated showed identical reassignment efficiencies in both Top10 and Top10/HisB-, confirming that reassignment efficiency in rich media is independent of the strain's ability to synthesize its own histidine.

All codon reassignment efficiencies represent the mean and standard deviation of the number of biological replicates indicated in the table.
